# Automatic detection of modal spacing (Yellott's ring) in adaptive optics scanning light ophthalmoscope images

**DOI:** 10.1111/opo.12070

**Published:** 2013-05-13

**Authors:** Robert F Cooper, Christopher S Langlo, Alfredo Dubra, Joseph Carroll

**Affiliations:** 1 Department of Biomedical Engineering, Marquette UniversityMilwaukee, USA; 2 Department of Cell Biology, Neurobiology and Anatomy, Medical College of WisconsinMilwaukee, USA; 3 Department of Ophthalmology, Medical College of WisconsinMilwaukee, USA; 4 Department of Biophysics, Medical College of WisconsinMilwaukee, USA

**Keywords:** adaptive optics, photoreceptor, repeatability, retinal imaging

## Abstract

**Purpose** An impediment for the clinical utilisation of ophthalmic adaptive optics imaging systems is the automated assessment of photoreceptor mosaic integrity. Here we propose a fully automated algorithm for estimating photoreceptor density based on the radius of Yellott's ring.

**Methods** The discrete Fourier transform (DFT) was used to obtain the power spectrum for a series of images of the human photoreceptor mosaic. Cell spacing is estimated by least-square fitting an annular pattern with a Gaussian cross section to the power spectrum; the radius of the resulting annulus provides an estimate of the modal spacing of the photoreceptors in the retinal image. The intrasession repeatability of the cone density estimates from the algorithm was evaluated, and the accuracy of the algorithm was validated against direct count estimates from a previous study. Accuracy in the presence of multiple cell types and disruptions in the mosaic was examined using images from four patients with retinal pathology and perifoveal images from two subjects with normal vision.

**Results** Intrasession repeatability of the power spectrum method was comparable to a fully automated direct counting algorithm, but worse than that for the manually adjusted direct count values. In images of the normal parafoveal cone mosaic, we find good agreement between the power-spectrum derived density and that from the direct counting algorithm. In diseased eyes, the power spectrum method is insensitive to photoreceptor loss, with cone density estimates overestimating the density determined with direct counting. The automated power spectrum method also produced unreliable estimates of rod and cone density in perifoveal images of the photoreceptor mosaic, though manual correction of the initial algorithm output results in density estimates in better agreement with direct count values.

**Conclusions** We developed and validated an automated algorithm based on the power spectrum for extracting estimates of cone spacing, from which estimates of density can be derived. This approach may be used to estimate cone density in images where not every single cone is visible, though caution is needed, as this robustness becomes a weakness when dealing with images from patients with some retinal diseases. This study represents an important first step in carefully assessing the relative utility of metrics for analysing the photoreceptor mosaic, and similar analyses of other metrics/algorithms are needed.

## Introduction

Adaptive optics (AO) retinal imaging systems permit direct visualisation of the rod and cone photoreceptor mosaics.^1^,^2^ Central to the clinical application of this imaging capability is having robust methods for analysing images of the photoreceptor mosaic, and there are a number of well-defined metrics derived for use on histological specimens that have been translated to the analysis of AO retinal images.^4^,^5^ Currently used metrics within the ophthalmic AO community require identification of cells within the region of interest, and include photoreceptor density,^7^ Voronoi-based analyses of mosaic geometry,^8^–^9^ the histogram-based density recovery profile,^10^ and the calculation of average inter-cell spacing.^11^ Given the expanding clinical applications for AO imaging,^12^–^15^ and the emergence of clinical prototypes,^16^,^17^ it is important that the relative merit of these metrics is objectively demonstrated to facilitate their use in comparative and prospective clinical studies.

Impeding progress in these efforts is the subjectivity of current cone identification processes in AO retinal images. Garrioch *et al*. recently quantified the repeatability of an automated algorithm for identifying individual cone photoreceptors,^19^ though similar inspection of other approaches is lacking. Metrics derived from directly counting the cells work well in images where every cell is resolvable; however in images of lower quality where some cells may not be visible, the accuracy of these methods could be significantly diminished. It is possible to avoid cone identification altogether by examining images of the photoreceptor mosaic in the frequency domain. Originally observed by Yellott,^20^–^21^ the Fourier transform of a cone mosaic image has an annular appearance. As described by Coletta and Williams,^22^ and as adopted here, the radius of this annulus corresponds to the modal frequency of the cone mosaic, and the reciprocal of this modal frequency is defined as the modal spacing of the cones in the original image. Numerous investigators have used this relationship to extract estimates of cone spacing from images of the living cone mosaic obtained using laser interferometry,^23^ fundus photography,^24^ and AO fundus photography.^1^ Derivation of an estimate of photoreceptor density from such modal spacing values requires some assumptions about the underlying geometry of the mosaic. Here, we use the commonly adopted assumption that the human cone mosaic is arranged in a regular triangular lattice.^22^–^25^ Extraction of the modal frequency has been a highly subjective process, thus limiting the widespread implementation of the technique.

Here we propose an algorithm for estimating modal cone spacing (and from this, cone density) from images of the photoreceptor mosaic using automated identification of the modal frequency in the power spectrum. We validate this algorithm against direct-count estimates of density using images of the normal parafoveal cone mosaic. To provide further assessment of the algorithm, we examined normal images of the perifoveal rod and cone mosaics as well as images of disrupted parafoveal cone mosaics from individuals with retinal disease.

## Methods

### Human subjects

All experiments were performed in accordance with the Declaration of Helsinki and study protocols were approved by the institutional research boards at the Medical College of Wisconsin and Marquette University. Parafoveal images from twenty-one subjects (13 males and eight females, age 25.9 ± 6.5 years) with no vision-limiting pathology were used from a previous study.^19^ Additionally, four subjects with retinal disease were recruited including a subject with retinitis pigmentosa (female, age 46 years), a subject with red-green colour blindness caused by an LVAVA mutation (male, age 15 years),^26^ a subject with red-green colour blindness caused by an LIAVA mutation (male, age 36 years),^7^ and a subject with photoreceptor disruption with an unknown aetiology (male, age 11 years). Two female subjects with normal vision (ages 23 and 27 years) were also recruited for this study, from whom images of the perifoveal photoreceptor mosaic were acquired. All subjects provided written informed consent after explanation of the nature and risks of the study. Axial length measurements were obtained using a Zeiss IOL Master (http://www.meditec.zeiss.com/iolmaster).

### Photoreceptor image acquisition and processing

A previously described AO scanning light ophthalmoscope (AOSLO) was used to image each subject's photoreceptor mosaic.^2^ The wavelength of the super luminescent diode used for retinal imaging was 775 nm. The system's pupil used for imaging was 7.75 mm in diameter and we estimate that the confocal pinhole of our system was about one Airy disk diameter. Image sequences of 150 frames were recorded at each retinal location, and the retinal area scanned was 0.96 × 0.96°. Intra-frame distortion due to the sinusoidal motion of the resonant optical scanner was estimated from images of a Ronchi ruling and removed by resampling each frame of the raw video over a square pixel grid. After desinusoiding, a reference frame with low distortion due to eye motion was manually selected from each image sequence for subsequent registration using a strip-based registration method.^27^ Each frame was divided into strips and each strip was registered against the reference frame by finding the relative position that maximises the normalised cross-correlation between them.^27^ The registered frames were then averaged to create a single high signal-to-noise ratio image for each image sequence.

As mentioned above, two datasets were used in this study. The normative dataset from Garrioch *et al*. consisted of images from four separate locations approximately 0.65° from centre of fixation.^19^ The four locations were imaged in a random order, with the subject remaining positioned on the chin/forehead rest for each set of image sequences. This procedure was repeated 10 times for each subject, with a short break in between each set, resulting in a total of 840 images (21 subjects, four locations per subject, 10 images per location). Garrioch *et al*. analysed the central 55 × 55 μm portion of each image,^19^ and we did the same. Due to individual differences in ocular magnification, the number of image pixels subtended by the 55 × 55 μm sampling window was variable across subjects (ranging from 120 to 148 pixels). The second dataset included images collected from four subjects with retinal disease at approximately 0.65° from centre of fixation and perifoveal images (about 10° temporal to fixation) from the two normal female subjects. For analysis, all photoreceptor images were transformed to a logarithmic intensity scale.

### Detecting Yellott's ring

The proposed algorithm is based on feature extraction and detection using pattern matching. To begin, the photoreceptor image was transformed into this using the discrete Fourier Transform (DFT), and this image was resampled to five times its size using bicubic interpolation.^1^

**Figure 1:**
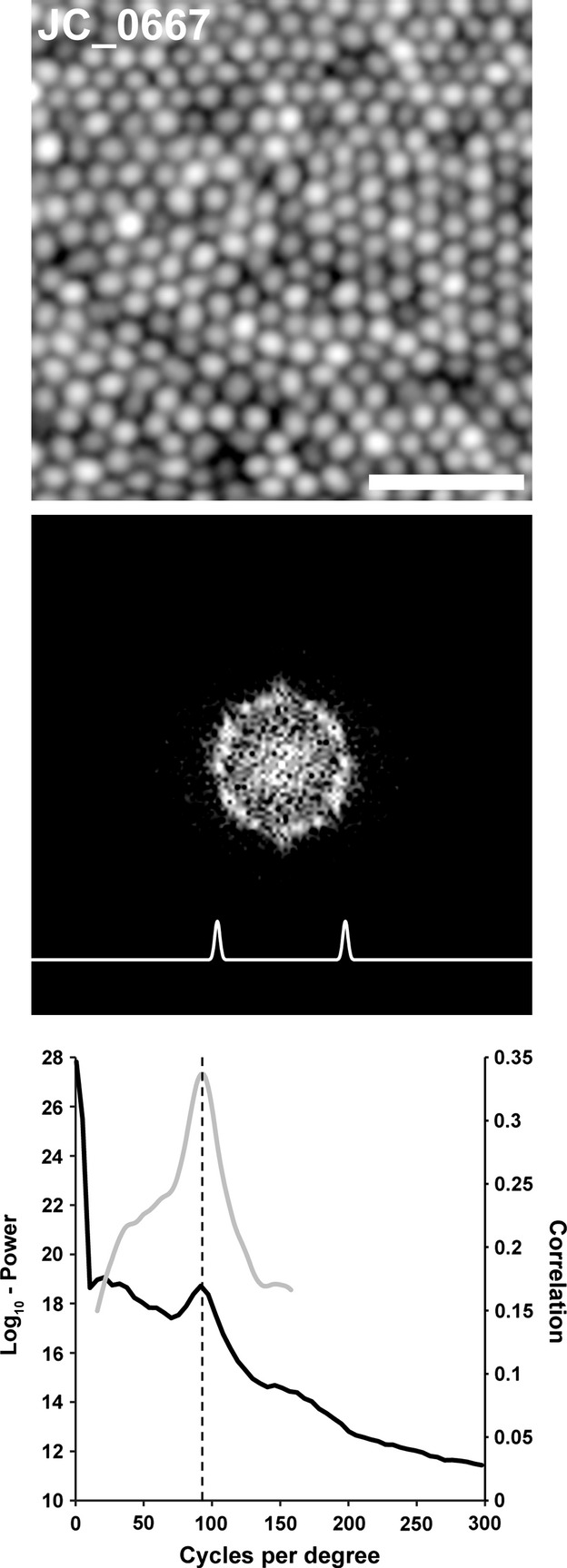
Extracting cone density from the power spectrum. Shown is an exemplar parafoveal cone photoreceptor image (log_10_-display, 0.5° Temporal and 0.5° Superior from fixation). Scale bar is 20 μm. The middle panel shows the 2D log_10_-power spectrum of the photoreceptor image in the top panel. Due to the highly regular mosaic in the image, the hexagonal packing of the photoreceptors is visible in the Fourier domain as a ring with peaks in a hexagonal pattern. The trace shown below the power spectrum ring represents the cross-section of the template whose radius was adjusted to best fit the power spectrum. The lower panel shows a plot of the radial average of the log_10_-power spectrum (*solid black line*) as well as a plot of the correlation function (*solid grey line*). The vertical dashed line indicates the peak of the correlation function automatically determined by the algorithm, which corresponds well to the visible peak in the radial average of the log_10_-power spectrum. This spacing corresponds to a density of 90 332 cones per mm^2^, which is close to the direct count value for this mosaic (86 344 cones per mm^2^).

The power spectrum was calculated as the log_10_ of the square of the absolute value of the DFT image (*Figure *1). Next, we created an annular template with a Gaussian profile centred on the power spectrum with a standard deviation of 7.5 cycles per degree, the cross-section is shown in *Figure *1. The cross-sectional width of Yellott's ring varies, in part, as a function of irregularity in packing geometry – the power spectrum from more irregularly packed mosaics will have a ring with a wider cross-sectional profile than that from more uniformly packed mosaics. Looking at previously published normative cone spacing values,^19^ irregularity in cone spacing would correspond to a standard deviation of about 5.5 cycles per degree in the frequency domain. As other factors, such as local variation in iso-orientation contours will also broaden the cross-sectional profile, we relied on empirical observations^28^ to set the standard deviation at 7.5 cycles per degree. While this parameter is adjustable, it was fixed for the present analysis. Normalised cross-correlation using Pearson's correlation coefficient was performed between the power spectrum and annuli of varying sizes to maximise the correlation between the pattern and the image. Considering physiological limits of the axial length of the human eye,^29^ as well as previously reported rod and cone density values for normal eyes,^30^ the radius of the annulus was allowed to vary from 15 to 160 cycles per degree, enabling detection of all physiologically plausible cell spacing values for this cohort.

### Intrasession repeatability of power spectrum derived density estimates

In order to assess the intrasession repeatability of cone density estimates derived with the algorithm, we assessed the power-spectrum derived cone density for all 840 normal parafoveal images. The power spectrum-derived spacing values were converted to density, *D*, in cells per mm^2^, using the approximation described in *equation* 1 (below), where *s* is the modal spacing in cycles per degree and *M* is the retinal magnification in mm per degree.^22^ It is important to note that this assumes the cells are arranged in a triangular crystalline mosaic. For a 24 mm axial length eye, the magnification is 0.291 mm per degree, and we estimated the magnification for each image using a linear scaling based on each subject's measured axial length.


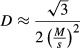
(1)

The repeatability measures were based on the within-subject standard deviation, *s*_*w*_, as described by Bland and Altman.^31^ To estimate *s*_*w*_, we first calculated the standard deviation of the repeated measures for each subject and then squared this to get the variance for each subject. The square root of the average variance for the 84 image sets (four per subject, 21 subjects) gives *s*_*w*_, and the repeatability is defined as *s*_*w*_ multiplied by 2.77. The 95% confidence interval for repeatability is given by *equation *2, where *n* is the number of subjects and *m* is the number of observations for each subject.^32^


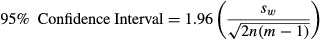
(2)

We compared these repeatability estimates to those previously reported for an automated cone counting algorithm (automated and automated with manual correction).^19^ Intrasession repeatability was expressed in cones per mm^2^ as well as a percentage of the mean value.

### Assessing the agreement between direct count and power spectrum derived density estimates

To validate the performance of this method, we examined the agreement between direct count density estimates and those derived from the power-spectrum method in the image set previously published by Garrioch *et al*.^19^ In this previous study, the direct-count density was measured over the central 55 × 55 μm portion of each image, and was obtained using the automated cone counting algorithm with manual correction. As we have repeated measures for both the direct count algorithm and the power-spectrum method, we can utilise all of the data to compare the agreement between the methods. While the details of this statistical approach have been provided in detail,^33^–^34^ we provide a brief overview here for this particular analysis. First, the average within-subject standard deviation is calculated for each method alone, as described above, denoted 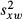 and 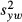. The mean difference between within-subject means is 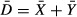. The variance of the differences between the within-subject means is given as 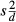. The number of observations on each subject by each method is given by *m*_x_ and *m*_y_. The adjusted variance of differences is then given by *equation *3.


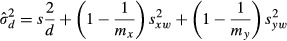
(3)

The 95% limits of agreement between the two methods is given by 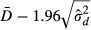 and 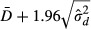. This comparison can be represented using a Bland-Altman plot, which plots the difference between the power spectrum derived density estimate and the direct count density against the mean of the two values, 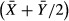. We also examined the agreement between the power spectrum method and direct count method in examples of non-uniform mosaics – parafoveal images in four patients with retinal pathology and two perifoveal images containing both rod and cone photoreceptors.

## Results

### Intrasession repeatability of power-spectrum derived estimates of cone density

We found that the algorithm had an average intrasession repeatability of 4953 cones per mm^2^ (95% CI = 4772 – 5133 cones per mm^2^). This corresponds to an intrasession repeatability of 6.7%. This means that the difference between any two measurements on the same subject would be less than 4953 cones per mm^2^ (or 6.7%) for 95% of observations with this algorithm. The measurement error, or expected difference between a measurement and the true value, was calculated to be 3504 cones per mm^2^. These statistics are summarised in *Table *1. This intrasession repeatability is comparable to that reported by Garrioch *et al*. who assessed the repeatability of cone density measurements using a fully automated direct count algorithm.^19^ Examination of their data reveals a repeatability of 4829 cones per mm^2^ (95% CI = 4653 – 5005 cones per mm^2^), or 6.4%.^2^

**Table 1:** Intrasession repeatability of cone density measurements derived from the power spectrum spacing

Fixation location	Mean density (cones per mm^2^)	Measurement error (cones per mm^2^)	Repeatability (cones per mm^2^)	95% CI for repeatability (cones per mm^2^)	Repeatability (%)	95% CI for repeatability (%)
Bottom left	72 712	2278	3219	3102–3336	4.4	4.3–4.6
Bottom right	71 555	2902	4102	3953–4251	5.7	5.5–5.9
Top left	77 224	3479	4917	4738–5096	6.4	6.1–6.6
Top right	74 129	5358	7573	7297–7849	10.2	9.8–10.6
Average	73 905	3504	4953	4772–5133	6.7	6.4–6.9

However, as shown by Garrioch *et al*., the use of manual correction of the automated density estimates resulted in improved repeatability of 2123 cones per mm^2^ (95% CI = 2046 – 2200 cones per mm^2^), or 2.7%.

### Comparison of direct count and power-spectrum-derived estimates of density

**Figure 2:**
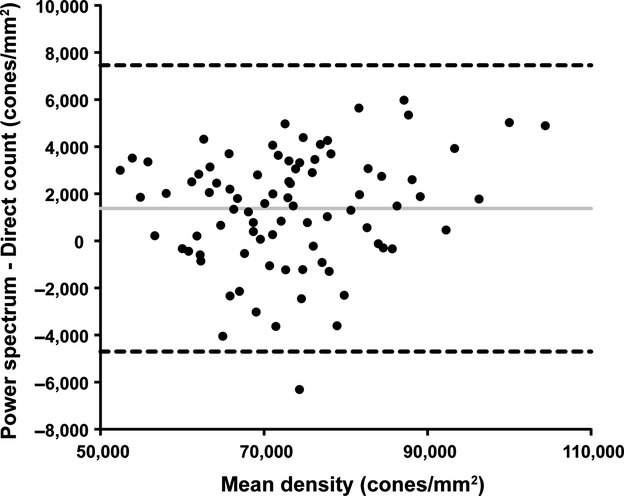
Bland-Altman plot of cone density derived from the direct count algorithm (with manual correction) and from the power-spectrum algorithm presented here. Solid lines: average mean difference (1377 cones per mm^2^); dotted lines: 95% confidence limits of agreement.

Despite having worse repeatability, the average cone density from the power-spectrum derived method (73 905 cones per mm^2^, *Table *1) was similar to the average cone density from the direct count with manual correction method (72 528 cones per mm^2^). Both of these values are greater than the average cone density reported for this data set using the fully automated direct count algorithm (68 535 cones per mm^2^).^19^ To quantify the agreement between the cone density from the power-spectrum derived method and that from the direct count with manual correction method, we created a Bland-Altman plot (*Figure *2). In the normative subset of images from Garrioch *et al*.,^19^ we found a difference, 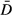, of less than 2%, with the power spectrum-derived density estimates being on average 1377 cones per mm^2^ greater than the direct-count measurements (*Figure *2). As this is comparable to the measurement error of either method, we consider this to represent good agreement between the methods. The adjusted variance of differences, 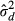, was calculated using *equation *3 and the 95% limits of agreement were found to be ± 6079 cones per mm^2^, and are represented by the dashed lines in *Figure *2. Overall, the differences do not vary systematically over the range of cone density measurements.

**Figure 3:**
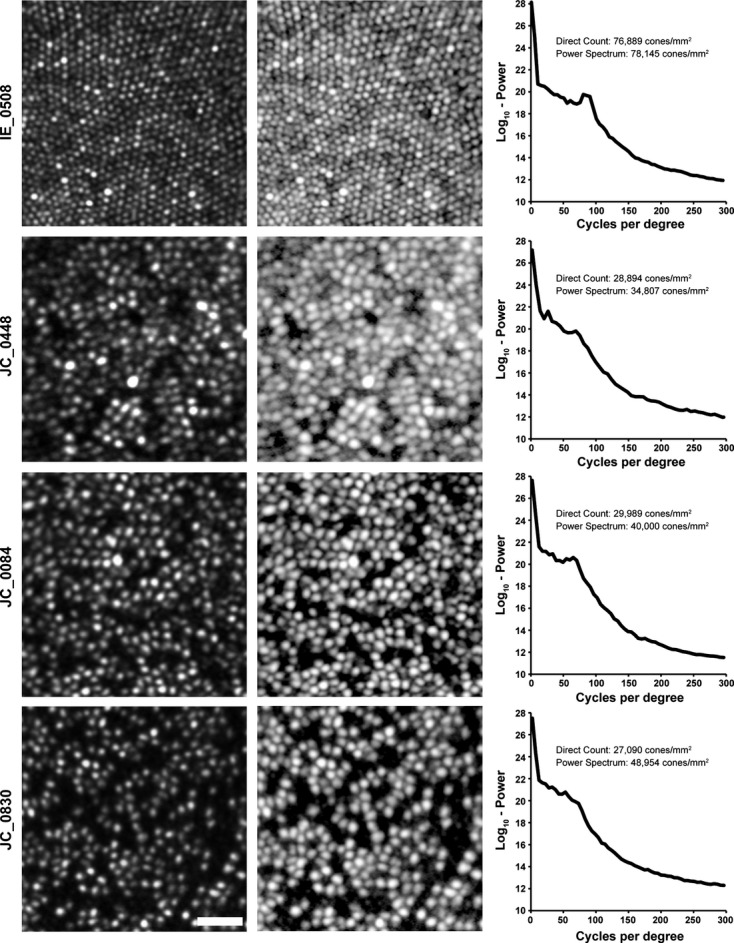
We examined power-spectrum derived estimates of cone density in patients with various pathologies. Shown in the left column are images from a subject with retinitis pigmentosa (IE_0508), a subject with red-green colour blindness caused by an LVAVA mutation (JC_0448),^26^ a subject with red-green colour blindness caused by an LIAVA mutation (JC_0084),^7^ and a subject with photoreceptor disruption with an unknown aetiology(JC_0830). Scale bar is 20 μm. Shown in the middle column is the log_10_-display of each image, with the radial average of the 2D log_10_-power spectrum shown in the right panel. As can be seen in the cone density values, there is disagreement in the mosaics with disrupted cone mosaics, with the power-spectrum derived density overestimating the actual density of the image.

We next sought to assess the agreement between these methods in individuals with retinal disease. In a patient with retinitis pigmentosa having a complete and contiguous parafoveal cone mosaic (*Figure *3, IE_0508), there is good agreement (76 889 cones per mm^2^ for direct count vs 78 145 cones per mm^2^ for power-spectrum derived). However, in patients with patchy disruption of the cone mosaic (JC_0448, JC_0084, JC_0830) we observed worse agreement with the power-spectrum derived value overestimating the direct-count density by 20-81% (*Figure *3). As the conversion of modal spacing in the power spectrum to density assumes a complete mosaic, this insensitivity to cell loss would invariably result in overestimation of the real density.

**Figure 4:**
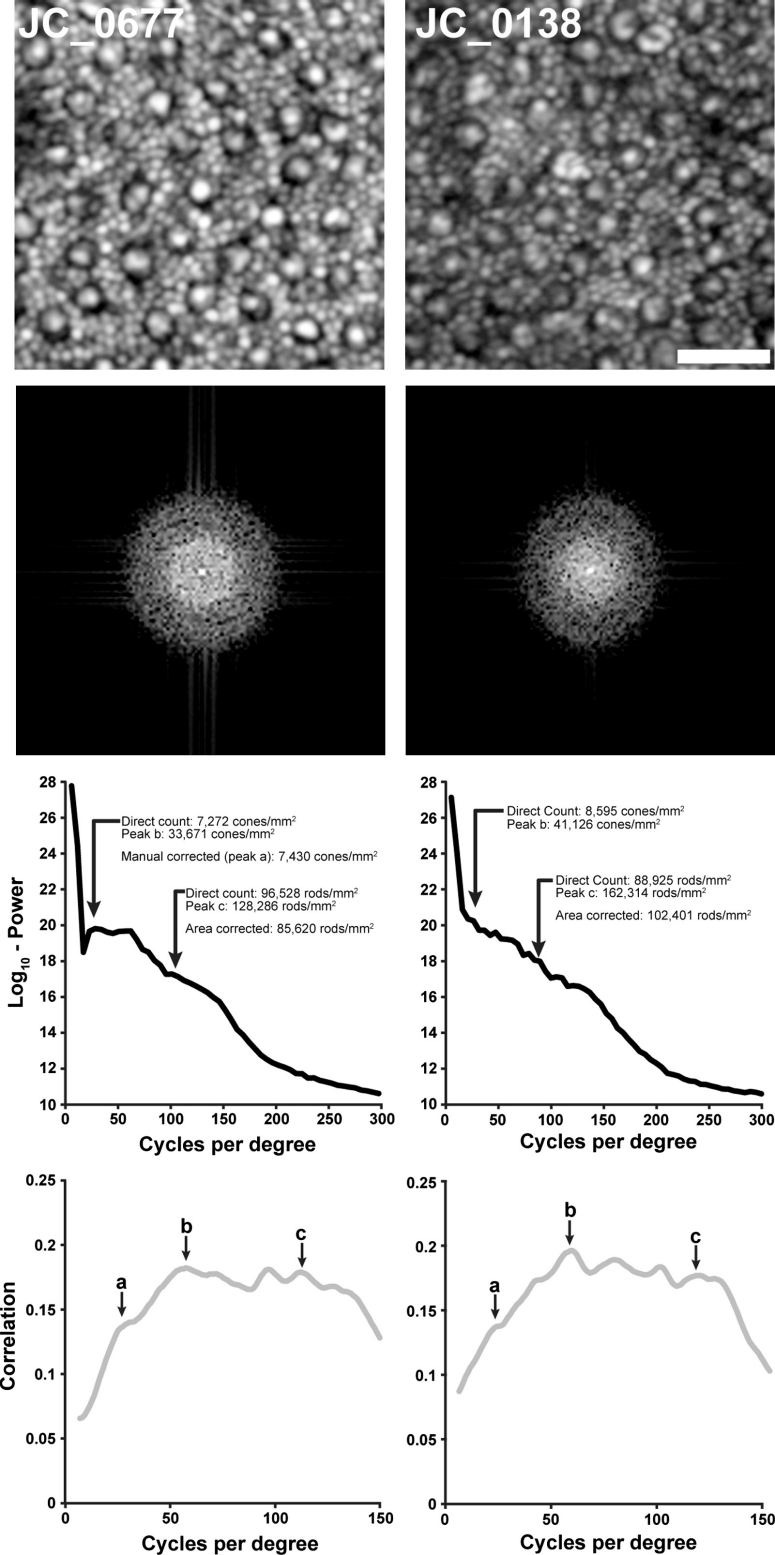
Estimating density in perifoveal images containing rod and cone photoreceptors. Shown are two retinal images (log_10_-display) from about 10 degrees temporal to fixation (scale bar is 20 μm), along with the 2D log_10_-power spectrum for each image. In these images, the automated power-spectrum derived density estimates overestimate the direct count density values for both the cone and rod mosaics. The presumed cone peak in the correlation plot (*peak b*) actually corresponds to the size of the cones themselves, not their modal spacing. The subtle peak on the ascending limb of the correlation plot (*peak a*) corresponds to cone spacing, using this spacing to estimate density yields values in better agreement with the direct count estimates. Using this estimate of cone density together with an estimate of the area of each cone from peak b and the modal spacing of the rod mosaic (*peak c*), it is possible to estimate the number of rods displaced by the cone mosaic and derive a corrected estimate of rod density. This approach yields rod density values in better agreement with the direct count values. Note that the x-axes of the lower plots are different scales.

As the resolution of ophthalmic AO instruments has improved, it is now possible to resolve rods as well as cones in images of the perifoveal photoreceptor mosaic.^35^,^36^ The presence of two distinct mosaics within a single image also compromises the accuracy of power-spectrum derived estimates of density. As shown in *Figure *4, density estimates based on the cone and rod modal spacing detected by the algorithm overestimate the direct count densities. However in both cases, the power spectrum contains information that can be used to correct the density estimates. In these images, most perifoveal cones have a bright central reflective core surrounded by a dark ring. We previously hypothesised that this dark ring represented the extent of the cone inner segment.^38^ It is this aspect of the cone profile that generates the dominant low frequency structure detected by the algorithm (peak b in *Figure *4). The spatial frequency of this peak in the two mosaics in *Figure *4 corresponds to a structure of 7.5 μm and 7.6 μm in diameter, consistent with previous histological estimates of cone inner segment diameter (about 7.8 μm) at this eccentricity for the temporal retina.^39^ There is a second weaker peak in the power spectrum that can be seen in the plot of the correlation function (peak a in *Figure *4). This manually identified peak corresponds to a cone density of 7430 cones per mm^2^ and 8355 cones per mm^2^ for the two mosaics in *Figure *4, much closer to the direct count estimates of 7272 cones per mm^2^ and 8595 cones per mm^2^, respectively.

On first glance, the rod density estimates calculated from the power-spectrum derived spacing are 1.5 times greater than the direct count density (*Figure *4). However, as stated in the methods, the derivation of an estimate of density from the modal spacing from the power spectrum assumes that the objects in the image are contiguous and periodic. In these perifoveal images, the cones disrupt the contiguity of the rod mosaic and thus the area occupied by the cones needs to be corrected for in order to extract an accurate estimate of rod density from the modal spacing. Using the cone density estimate derived from the cone spacing (peak a in *Figure *4) and an estimate of cone area based on the cone size (peak b in *Figure *4), together with the estimated rod spacing from the power spectrum (peak c in *Figure *4), we can estimate the number of rods displaced by the cones in the image. This provides a corrected power-spectrum derived estimate of 85 620 rods per mm^2^ and 102 401 rods per mm^2^ which is in better agreement with the direct count estimates of 96 528 rods per mm^2^ and 88 925 rods per mm^2^, respectively. While the need for manual selection of the additional peaks in these power spectra severely limits the clinical utility of this approach, our analysis provides a good starting point to examine the interplay between the rod and cone submosaics in the frequency domain image.

## Discussion

We developed and tested an automated algorithm for extracting estimates of cone and rod density from the power spectrum. In AOSLO images of the normal parafoveal cone mosaic, the power spectrum-derived density estimates showed good agreement with direct-count estimates (an average bias of 1377 cones per mm^2^, or 1.9%), and the algorithm's average repeatability was 6.7%. This repeatability is comparable to that reported for a fully automated direct count algorithm, however the power-spectrum derived density estimates are actually closer to the true value (direct count + manual correction) than are the estimates obtained by the fully automated direct count algorithm. Of course in high-quality images where every cell is visible, a direct count approach would be preferred as it offers the opportunity to assess additional metrics such as the packing geometry of the mosaic, which requires a 2D map of the cell positions. However, in images where not every cell is visible or that are of generally poorer quality, direct counting may prove more unreliable. Thus the power spectrum method may in fact be preferred for deriving estimates of cell density. Examination of additional datasets from other AO devices is needed to test this concept. As illustrated in *Figure *3, this robustness (*i.e*., insensitivity to not every cell being visible) actually becomes a liability when analysing mosaics from diseased retinae with cells that have degenerated. This would greatly diminish the clinical utility of the power spectrum method.

There are additional limitations to the method proposed here. First, the estimation of density from the power spectrum spacing relies on the assumption of a crystalline triangular mosaic.^22^ While in normal mosaics this presumption of regularity holds,^8^–^41^ it fails in mosaics of patients with retinal degeneration. As shown in *Figure *3, this would limit the accuracy of density estimates from disrupted mosaics. A second limitation is that the power spectrum contains information about the object profile as well as the spacing of the objects in the image. Given that the cone profile varies with eccentricity, focal plane, and with disease,^15^–^43^ disentangling the contribution of the cone profile will be difficult. However, it was recently shown that the cone profile can be manipulated through the use of annular pupils,^44^ and this may provide a way to tease apart the relative impact of the cone profile on the power spectrum.

There are additional limitations to the method proposed here. First, the estimation of density from the power spectrum spacing relies on the assumption of a crystalline triangular mosaic.^22^ While in normal mosaics this presumption of regularity holds,^8^–^41^ it fails in mosaics of patients with retinal degeneration. As shown in *Figure *3, this would limit the accuracy of density estimates from disrupted mosaics. A second limitation is that the power spectrum contains information about the object profile as well as the spacing of the objects in the image. Given that the cone profile varies with eccentricity, focal plane, and with disease,^15^–^43^ disentangling the contribution of the cone profile will be difficult. However, it was recently shown that the cone profile can be manipulated through the use of annular pupils,^44^ and this may provide a way to tease apart the relative impact of the cone profile on the power spectrum.

Given the continued improvements in retinal image quality combined with development of additional algorithms for automatically identifying photoreceptors in AO retinal images, the utility of the power spectrum method may not be in computing cell density. However, the agreement between the power-spectrum derived density and that from an automated algorithm in the complete, continuous mosaics analysed here may offer a sort of screening tool for automatically examining images in a clinical setting. For example, if used in conjunction with an automated cone identification algorithm, the power spectrum method could be used to flag images that require manual inspection, based on the magnitude of disagreement between the methods. Alternatively, the power spectrum method could be integrated into future algorithms to instruct them as to the modal spacing of the objects to be detected.

While this study provides a detailed examination of the relationship between two particular metrics for describing the photoreceptor mosaic, similar analyses of alternative methods for objectively characterising the photoreceptor mosaic are needed. As clinical applications of AO retinal imaging expand, it is important to understand the information provided by various mosaic metrics to converge on approaches that are both clinically practical and relevant.

## Disclosure

The authors report no conflicts of interest and have no proprietary interest in any of the materials mentioned in this article.

## Notes

1 Resampling the Fourier domain image to 1 cycle per degree corresponds to densities of between 293 and 3122 cones per mm^2^ (depending on the spacing of the mosaic being measured and the axial length of the eye). This sampling is adequate for comparing the cone density estimates from the power spectrum method against those obtained from the direct counting method (given that 1 cone in the direct count method in a 55 × 55 μm image would correspond to a density of 330 cones per mm^2^).

2 Note that the exact values reported here for the Garrioch *et al*. study differ slightly from their published intrasession repeatability and measurement error. They calculated *s*_*w*_ by averaging the standard deviation of the repeated measures for each subject, rather than averaging the variance of the repeated measures for each subject and then taking the square root of that value, which emphasises the contribution of outliers and would provide a more conservative estimate of *s*_*w*_ (which is the approach we chose here). The difference is not large, for example, the original reported measurement error for the fully automated cone identification algorithm was 3084 cones per mm^2^, whereas our calculation gives a value of 3417 cones per mm^2^.
